# Evaluation of the clinical assessment scale for autoimmune encephalitis (CASE) in a retrospective cohort and a systematic review

**DOI:** 10.1007/s10072-024-07642-1

**Published:** 2024-06-11

**Authors:** Eva Soellradl, Tim J. von Oertzen, Judith N. Wagner

**Affiliations:** 1 Kepler University Hospital, Johannes Kepler University, Linz, Austria; 2 University Hospital Würzburg, Würzburg, Germany; 3 Department of Neurology, Evangelisches Klinikum Gelsenkirchen, Teaching Hospital University Duisburg-Essen, Gelsenkirchen, Germany

**Keywords:** Autoimmune encephalitis, Clinical assessment, CASE score, Modified Rankin scale

## Abstract

**Background:**

Autoimmune encephalitis (AE) poses significant challenges in clinical management, requiring effective monitoring tools for therapeutic success and relapse detection. This study aims to assess the Clinical Assessment Scale in Autoimmune Encephalitis (CASE) as compared to the modified Rankin scale (mRS) in evaluating AE patients and to determine the real-world adoption of the CASE score.

**Methods:**

A retrospective cohort study was conducted on 20 AE patients, assessing clinical data including symptomatology, diagnostic findings, and therapeutic regimens. Furthermore, we performed a systematic review on the test performance criteria and the real-world use of the CASE score.

**Results:**

The CASE score showed a higher sensitivity in detecting clinical changes compared to the mRS, with a significant correlation between the two scales throughout the disease course (r = 0.85, p < 0.01). A systematic review of 150 articles revealed widespread adoption of the CASE score, especially in Asian populations, demonstrating high reliability and internal consistency.

**Discussion:**

Despite limitations such as retrospective design and small sample size, our findings underscore the CASE score's utility in both clinical practice and research settings. The CASE score emerges as a valuable tool for monitoring AE patients, offering improved sensitivity over existing scales like the mRS. Further validation studies in diverse populations are warranted to establish its broader applicability and inform future therapeutic interventions.

## Introduction

With the first description of autoimmune encephalitis (AE) associated with antibodies against extracellular antigens in 2007, optimization of therapeutic approaches has aroused large interest. [1] The efficacy of early and determined immunotherapy has repeatedly been demonstrated [2, 3]. Hence, monitoring of therapeutic success and early recognition of relapses is essential.

The modified Rankin scale (mRS) has been developed to score global disability post-stroke [4]. Hence, non-motor symptoms such as psychiatric symptoms, epileptic seizures and language deficits are insufficiently represented. These symptoms feature prominently in AE. The clinical assessment scale for autoimmune encephalitis (CASE) score has been specifically devised for evaluation of these patients [5]. On a scale from 0 to 27, severity of nine motor and non-motor symptoms is specified.

Of the multitude of clinical scores that are developed, only a minor part enters into regular use in clinical practice, while others serve as instruments in scientific studies or disappear completely. On the basis of a cohort of or own patients and a systematic literature review, we aim to resolve the following issues:

- Does the CASE score display superior sensitivity to alterations of the clinical status in patients with AE compared to the mRS?
- Do CASE and mRS scores correlate?
- Has the CASE score been adopted in clinical practice and scientific research on AE?

## Methods

All patients with a ICD 10 diagnosis consistent with encephalitis who were treated at one of the participating centers (Kepler University Hospital Linz (Austria) – Department of Neurology 1, Kepler University Hospital Linz (Austria) – Department of Neurology 2) between January 1st 2007 and April 30th 2020 were screened. Included were those patients, who fulfilled the diagnostic criteria of autoimmune encephalitis:

- Clinical syndrome compatible with autoimmune encephalitis and detection of a well-characterized antibody
- In antibody-negative patients: fulfillment of the Graus-criteria of (at least) “probable” autoimmune encephalitis [6]
- Sufficient data to retrospectively evaluate the clinical course of the patient

Relevant data of the initial hospitalization as well as on further hospital stays and outpatient visits were extracted from the electronic patient file. These included type, duration and extent of clinical symptoms, laboratory results including CSF (cerebrospinal fluid) findings and antibody titers, imaging results and EEG (electroencephalography) characteristics, and therapeutic regimens. The patients’ symptoms were retrospectively scored at initial hospitalization, at hospital discharge, and on last follow-up available on the mRS and the CASE score. MSOffice was used to create figures.

Descriptive statistics were used. Correlation was investigated using the Pearson coefficient (r). The significance level was set to 0.05. Statistic analysis was performed by ES using Excel 2010. The study was approved by the ethics committee of Upper Austria.

The main outcome assessed via systematic review was real-world use of the CASE score in clinical practice. Secondary endpoints were inter- and intraobserver reliability, internal consistency, and correlation of CASE and mRS scores.

We performed a MEDLINE literature search using PubMed to identify all reports published between January 1, 2019 and January 18, 2024. The starting date was set to match the date of initial publication of the CASE score. The search term was ((((clinical) AND (assessment)) AND (scale)) AND (autoimmune)) AND (encephalitis). Other databases searched include the Clarivate/ Web of Science (search term “ALL = (Clinical Assessment Scale in Autoimmune Encephalitis)”), Cochrane Database and the ClinicalTrials.gov website.

Titles and abstracts of the reports obtained were screened for inclusion in the review using the following criteria: population with autoimmune encephalitis, patients were evaluated via the CASE score. Articles published in languages other than English, German, French or Spanish as well as duplicate studies, preclinical studies, editorials and reviews were excluded except for secondary search. No further exclusion criteria applied. Included were all case reports, case series, retrospective and prospective observational studies, and randomized controlled trials. A secondary search for other relevant articles was performed in the articles included after full-text analysis as well as in reviews on the topic.

## Results

We included a total of 20 consecutive patients (12 male). Median age at onset was 58 years (IQR 37 – 67 years). Diagnosis was autoimmune encephalitis with antibodies against LGI1 (n = 4), CASPR2 (n = 4), the NMDA receptor (n = 3), and the GABA_B_ receptor (n = 1). 8 patients were antibody-negative.

The most common symptom were epileptic seizures (n = 20) and cognitive disorders (n = 18). 75% of patients displayed MRI lesions, particularly affecting the mesio-temporal structures, 100% had alterations on EEG (epileptiform discharges, seizure patterns, regional slowing).

In 14 patients, therapy was initiated at our centers. The most frequently used first-line therapy was high-dose steroids (n = 13) and intravenous immunoglobulins (IVIG; n = 11). The most commonly used second-line therapy was rituximab (n = 3). Levetiracetam was the most prevalent antiseizure drug (n = 11), followed by lacosamide (n = 7), and valproate (n = 6). For 13 patients, data on the interval between onset of symptoms and initiation of anticonvulsive therapy and immunotherapy were available (median 52 days, IQR 4–161 days and median 61 days, IQR 6–242 respectively). Eight patients required ICU-treatment. Within a median follow-up period of 1117 days (IQR 364 – 3309 days), two patients experienced an encephalitis relapse.

**Fig. 1:**
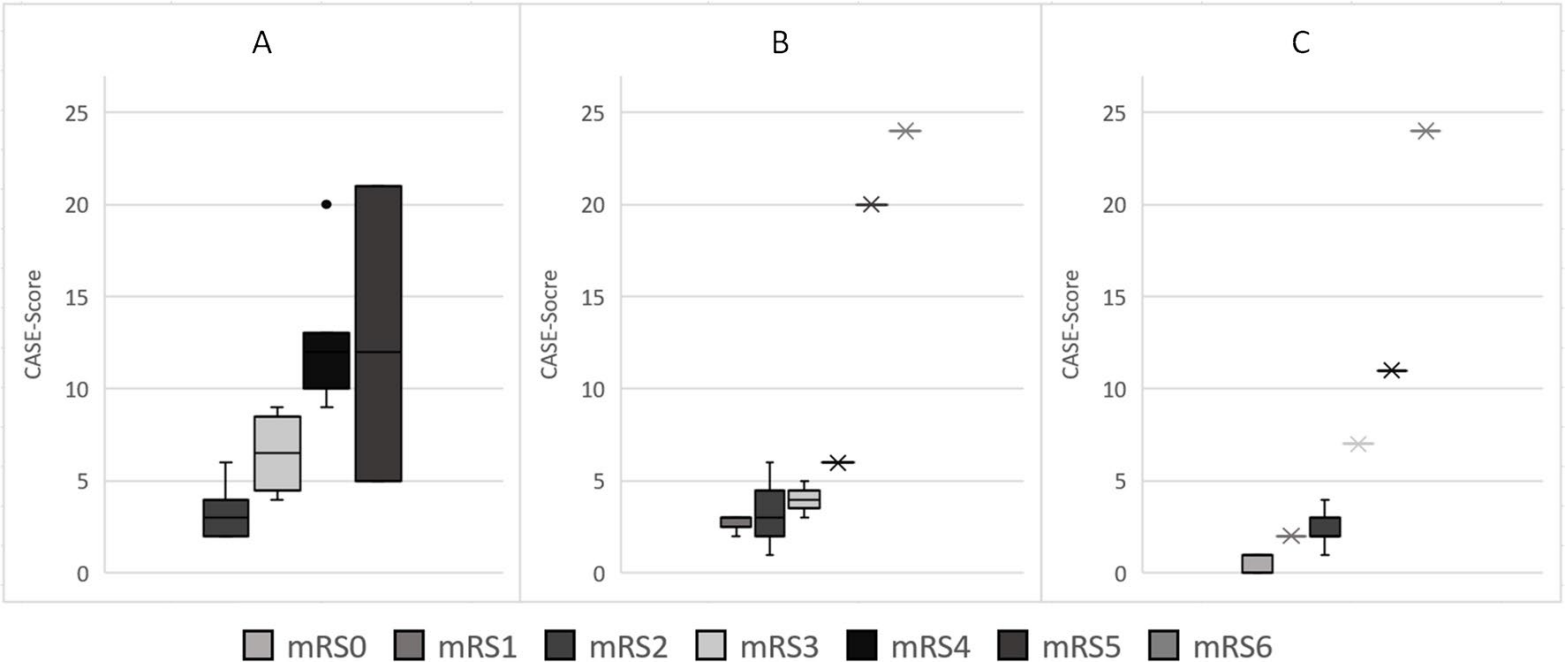
Distribution of CASE scores along the range of mRS scores at baseline (A), discharge (B), and last follow-up (C)

**Fig. 2:**
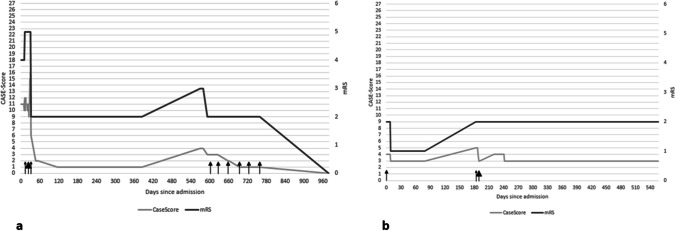
Evolution of the CASE and mRS score in a patient with anti-NMDAR AE (Fig. 2a) and in a patient with anti-LGI1 AE (Fig. 2b). Small arrows indicate iv high-dose steroid, large arrows iv immunoglobulin application. Medium arrows designate the time points of combined high-dose steroid and iv immunoglobulin therapy

During initial hospitalization, the CASE score changed in 15 patients and the mRS score in 11 out of 20 patients between admission and discharge. At last follow-up, all 20 patients had displayed a change in CASE scores, while only 15 had experienced changes in mRS score. Results of both scales showed a good correlation during the entire course of the disease (r = 0.85, p < 0.01). For the distribution of CASE and mRS scores at admission, discharge and last follow-up see Tables 1 and 2. Figure 1 shows the distribution of the CASE score at different mRS scores at initial hospitalization, discharge and last follow-up. For illustration purposes, the clinical and therapeutic course of two patients positive for anti-NMDAR receptor and anti-LGI1 antibodies is graphed in Figs. 2a and 2b. More frequent changes mirror more closely the clinical development of the patient thoughout the course of the disease.

**Table 1:** Number of patients with designated CASE score at admission, discharge and last follow-up

CASE Score	Hospital admission (n)	Hospital discharge (n)	Follow-up (n)
0	0	0	3
1	0	2	5
2	3	3	5
3	2	6	1
4	3	2	1
5	2	2	0
6	1	3	0
7	0	0	1
8	1	0	0
9	2	0	1
10	0	0	0
11	1	0	1
12	2	0	0
13	0	0	0
14	1	0	0
15	0	0	0
16	0	0	0
17	0	0	0
18	0	0	0
19	0	0	0
20	1	1	0
21	1	0	0
22	0	0	0
23	0	0	0
24	0	1	2

**Table 2:** Number of patients with designated mRS score at admission, discharge and last follow-up

Modified Rankin Scale	Hospital admission (n)	Hospital discharge (n)	Follow-up (n)
0	0	0	7
1	0	3	1
2	8	11	7
3	4	3	1
4	5	1	2
5	3	1	0
6	0	1	2

For 10 patients, serum antibody levels were determined at least three times in the same laboratory using commercially available cell-based assays. While individual cases displayed a good congruency of CASE score and antibody titer, we did not see this correlation throughout our cohort..

**Fig. 3:**
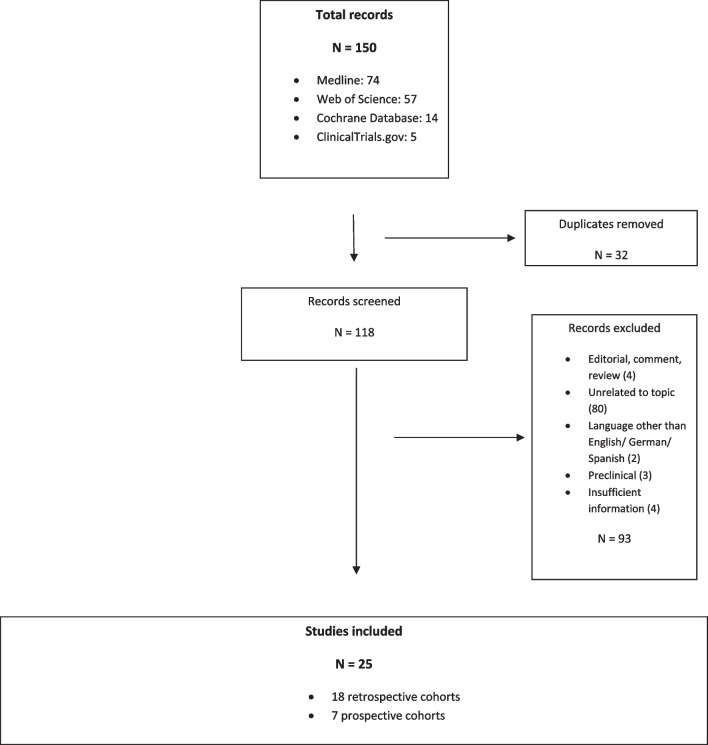
Flow-chart depicting the selection process of reports included in the review according to PRISMA guidelines

For the systematic review, we screened 150 articles. 25 of those were included: 18 retrospective cohort studies, seven prospective cohort studies (Fig. 3). These report on a total of 2003 subjects. Most populations investigated were adults (15 years and older). Four studies included only children, four more a mixed population. Five studies were specifically designed to develop, evaluate and/ or validate the CASE score. All of these were performed in an Asian population [5, 7–10]. Other aims include prognostication and risk prediction, correlation of outcome with paraclinical parameters, and therapeutic evaluation. Seven studies included only patients with NMDAR-antibodies, one each patients with LGI1-antibodies and antibody-negative AE, seven a cohort with antibodies directed against different neuronal surface structures, and nine a mixed population. Of note, the CASE score was developed on a rather heterogeneous cohort including patients with antibody-negative but probable AE, brainstem encephalitis and ADEM [5].

Reported inter-/intraobserver reliability range from 0.95/0.94 to 0.994/0.98, internal consistency from 0.825 to 0.92 (Cronbach alpha). mRS scores were reported in 24 studies, 10 of these calculate the correlation coefficient of CASE and mRS scores. The r-values in these reports range from 0.459 to 0.86. The lowest correlation was found in a cohort of children [10]. Four groups report the CASE/mRS correlation at different points in time (baseline and at least one follow-up). All but one found an increasing correlation at later data acquisition. The greatest difference of the r-value between baseline and last follow-up is 0.25 [11]. Geographic areas most frequently investigated include China (12; total number of subjects 1344), South Korea (5), and USA (4). One study took place in Germany, India, Austria, and France respectively.

## Discussion

The CASE score has become a well-accepted tool of clinical and therapeutic evaluation. While most reports on the validity of the CASE score published so far originate in Asian countries, we confirm these findings for a European population.

Although CASE score and mRS scale correlate reasonably well in most studies including ours, the CASE score has been shown to have a higher sensitivity for clinical changes than the mRS scale [7]. As it includes more symptoms that are frequent in and/or relatively specific to AE, it is more sensitive to changes of AE severity and more inert to clinical alterations secondary to other intercurrent conditions. These properties may account for the merely moderate correlation of mRS and CASE score in some cohorts. We report two illustrative examples for this divergence between both scores. The first one is a patient with anti-NMDAR encephalitis in whom the CASE score was more apt to detect therapeutic response. In a patient with LGI1 encephalitis, dementia caused an mRS score increase to 2 points irresponsive to immunotherapy. The CASE score, on the other hand, demonstrated a persistent improvement in the patient’s clinical status.

The greater sensitivity displayed by the CASE compared to the mRS score for alterations of the clinical state in AE is also reflected by the higher number of patients that exhibited a change of this score during initial hospitalization (75% vs. 55%). The results of our study coincide with a recent trial that demonstrated a change of CASE scores in 56.6% of patients between hospital admission and discharge. For the mRS, this was the case in only 33.6% [7].

Figure 1 illustrates the greater differentiation of the CASE compared to the mRS score, particularly for severe forms of the disease. It also demonstrates the favorable prognosis for most AE patients. Consequently, the small spread of CASE score values for higher mRS scores at discharge and follow-up may be due to the low number of cases in these subgroups. Alternatively, the data from our review suggest a better correlation of both scales at low scores, i.e. in patients displaying few symptoms and/or disability [11–13].

The lack of correlation between antibody levels and changes of the CASE score reflects the uncertain prognostic value of antibody titers [14, 15].

In summary, the CASE score demonstrates a high reliability and internal consistency that render it a valuable tool for evaluating AE patients in research and clinical practice. As such, it has become widely used in clinical practice only 5 years after it has been developed. However, larger validation studies in diverse ethnic populations with more homogenous disease entities are still lacking.

We recommend regular, ideally daily scoring of AE patients for monitoring of therapeutic success while hospitalized and on follow-ups. The CASE score also promises to be a powerful tool for future therapeutic trials. Shortcomings of this study include the retrospective approach, the low total number of patients and the heterogeneous cohort.

## Data Availability

Original data in anonymized form can be obtained from the corresponding author.
